# P-1525. Real-World Efficacy of Fosfomycin for Treatment of Cystitis Caused by *E. coli* Versus Non-*E. coli* Gram Negatives With High Rates of FosA

**DOI:** 10.1093/ofid/ofae631.1694

**Published:** 2025-01-29

**Authors:** Ryan C McCormick, Olivia Randazza, Michael E DeWitt, Mary Banoub, Alex D Taylor, Vera P Luther, Julia Christine Cook, John C Williamson

**Affiliations:** Prisma Health, Columbia, South Carolina; Atrium Health Wake Forest Baptist, Winston-Salem, North Carolina; Atrium Wake Forest Baptist Health/ Wake Forest University School of Medicine, Winston-Salem, North Carolina; Atrium Health Wake Forest Baptist Medical Center, Winston Salem, North Carolina; Atrium Health Wake Forest Baptist, Winston-Salem, North Carolina; Wake Forest University School of Medicine, Winston Salem, NC; Atrium Health Wake Forest Baptist Medical Center, Winston Salem, North Carolina; Atrium Health Wake Forest Baptist, Winston-Salem, North Carolina

## Abstract

**Background:**

Fosfomycin is an appealing cystitis treatment; however, guidelines recommend against fosfomycin for non-*E. coli* gram negatives due to reduced in vitro activity associated with FosA, an enzyme that hydrolyzes fosfomycin but does not always result in full resistance. These concerns have yet to be corroborated in efficacy studies. The purpose of this study was to determine if fosfomycin is an acceptable treatment for cystitis caused by non-*E. coli* gram negatives with high rates of FosA.

**Figure:**
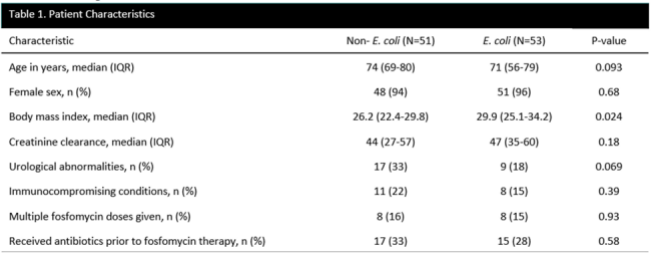

**Methods:**

This was a single-center, retrospective, cohort study evaluating the efficacy of fosfomycin for cystitis caused by *E. coli* versus non-*E. coli* gram negatives with high rates of FosA, including *K. pneumoniae*, *P. aeruginosa*, *K. aerogenes*, *K. oxytoca, M. morganii*, and *E. cloacae*. Patients with an inpatient or outpatient fosfomycin order to treat cystitis caused by these bacteria were included. Patients were excluded if there was a lack of cystitis symptoms, evidence of pyelonephritis, polymicrobial culture, or ≥ 48 hours of antimicrobial therapy prior to fosfomycin. The primary outcome was 30-day treatment failure defined as persistence or recurrence of cystitis symptoms with either a positive culture (same bacteria) or antibiotic re-treatment.

**Figure:**
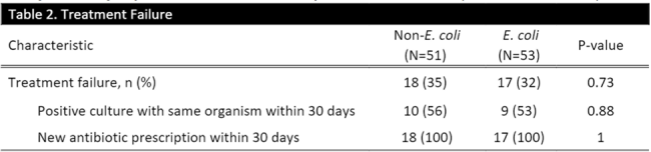

**Results:**

104 cases of cystitis were included in the study, 53 in the *E. coli* group and 51 in the non-*E. coli group*. Patient characteristics are presented in Table 1. The *E. coli* group had a higher median BMI; 26 (49%) vs 13 (25%) were obese (p=0.013). With 32 (63%) cases, *K. pneumoniae* was the most common non-*E*.*coli* and all but 10 cases were Enterobacterales. Treatment failure occurred in 17 (32%) of the *E*. *coli* group and 18 (35%) of the non-*E. coli* group (p=0.73) (Table 2). Among the failures, about half had a repeat positive culture, all failures received re-treatment, and median time to failure was 10 days for *E*. *coli* vs 7 days for non-*E*. *coli*. Figure 1 shows time to failure within groups, and Figure 2 shows failures by species.

**Figure:**
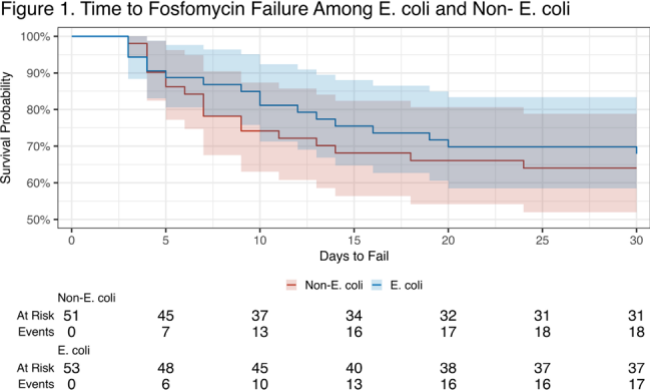

**Conclusion:**

These data suggest failure rates for fosfomycin are relatively high in all cases of gram negative cystitis, regardless of FosA. Presence of FosA may not be as clinically relevant as guidelines suggest when deciding to use fosfomycin for cystitis. Additional studies are needed to validate these findings.

**Figure:**
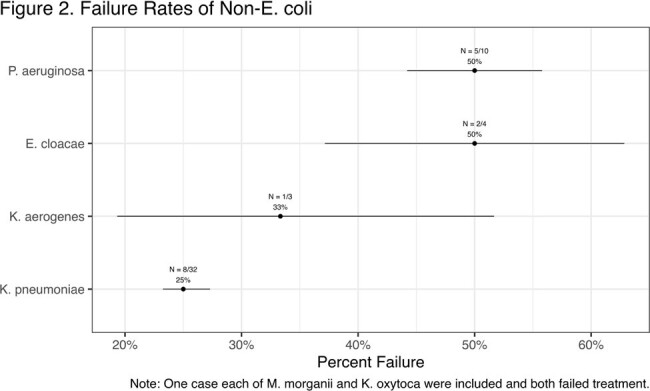

**Disclosures:**

**John C. Williamson, PharmD**, Armata Pharmaceuticals: Grant/Research Support|Blue Collar Vaccines and Therapeutics: Board Member|Blue Collar Vaccines and Therapeutics: Ownership Interest|Paratek Pharmaceuticals: Grant/Research Support|ST Pharm Co, Ltd: Grant/Research Support

